# Trichinellosis: A zoonosis that still requires vigilance

**DOI:** 10.1371/journal.pntd.0013944

**Published:** 2026-01-30

**Authors:** Ivana Mitic, Sasa Vasilev, Alisa Gruden-Movsesijan

**Affiliations:** Department for Immunology and Immunoparasitology, National Reference Laboratory for Trichinellosis, Institute for the Application of Nuclear Energy, University of Belgrade, Belgrade, Serbia; Institute of Continuing Medical Education of Ioannina, GREECE

## Abstract

Trichinellosis is a parasitic disease in humans caused by nematodes of the genus *Trichinella*, usually acquired through the consumption of raw or undercooked meat, mainly from pigs or wild animals, containing infective larvae. Although often overlooked, this disease has affected humans for centuries and continues to be a public health concern in many parts of the world, especially where food safety measures are inadequate. In this millennium, significant progress has been made in understanding the disease pathophysiology and host immune response, mainly through experimental studies in rodents, while human pathophysiology and immune response still await elucidation. This review integrates current knowledge on the biology, transmission, and manifestations of trichinellosis, as well as challenges in diagnosis, treatment, and prevention. The parasite’s life cycle involves an initial intestinal phase followed by systemic migration of larvae into striated muscles, where long-lasting cysts are established. The clinical presentation varies from mild gastrointestinal discomfort to severe systemic manifestations such as fever, periorbital edema, and muscle pain. In some cases, chronic symptoms, including fatigue and reduced muscle strength, may persist for years. The immune response is characterized by a Th2-skewed profile and prominent eosinophilia. Diagnosis relies on clinical signs and symptoms, epidemiological evidence of a common infection source, serological testing, and parasitological confirmation through detection of larvae in infected meat and identification of the *Trichinella* species. Preventive strategies include public education, controlled animal farming, and meat inspection. A deeper understanding of host–parasite interactions, chronic disease mechanisms, and species-specific differences remains essential for improving clinical management and guiding public health interventions.

**Methods:**

A comprehensive literature search was performed in PubMed, Scopus, Google Scholar, and Web of Science to identify publications on *Trichinella* and trichinellosis from 1990 through August 2025. The search strategy employed keywords such as “trichinellosis,” “*Trichinella*,” “*Trichinella* life cycle,” “trichinellosis pathophysiology,” “diagnosis,” “epidemiology,” “treatment,” and “control.” Only articles published in English were considered. Titles and abstracts were screened for relevance, and full-text articles were assessed to extract information on life cycle, epidemiology, pathophysiology, clinical manifestations, diagnostic approaches, therapeutic options, and prevention strategies. Additional references were identified by cross-checking the bibliographies of the included articles.

## Introduction

Infections caused by larvae of nematodes from the genus *Trichinella* have been reported worldwide, with human or animal documented cases in nearly 95 countries [1]. Infected animals remain asymptomatic, even when harboring high larval burden. In contrast, infection in humans leads to trichinellosis, a zoonotic disease reported in 55 countries [2]. *T. spiralis* is the main common cause of trichinellosis, although other *Trichinella* spp. such as *T. britovi*, *T. nativa*, *T. nelsoni*, *T. pseudospiralis*, *T. murrelli*, and *T. papuae* are also associated with human disease [3]. *Trichinella* spp. can be distinguished based on the presence or absence of collagenous capsule surrounding the nurse cell. Encapsulating species infect only mammals, while non-encapsulating species infect mammals, birds, and some reptiles [4].

## History

The modern scientific recognition of the parasite began in 1835 with the observation of “sandy diaphragm” by medical student Jim Paget during autopsy, which eventually led to the identification of the parasite and its presentation to the Royal Society. Richard Owen published the description of newly discovered parasite and named it *Trichina spiralis*. Over the following decades, key milestones were reached: the identification of *Trichinella* in pork and thereafter in other domestic and wild animals, elucidation of the life cycle, confirmation of its pathogenicity in humans, the establishment of the genus *Trichinella*, and development of diagnostic methods, including muscle biopsy and serological testing. These advances laid the foundation for our current understanding of trichinellosis as both a zoonotic and foodborne parasitic disease [5].

## Description and the life cycle of the parasite

**Fig 1:**
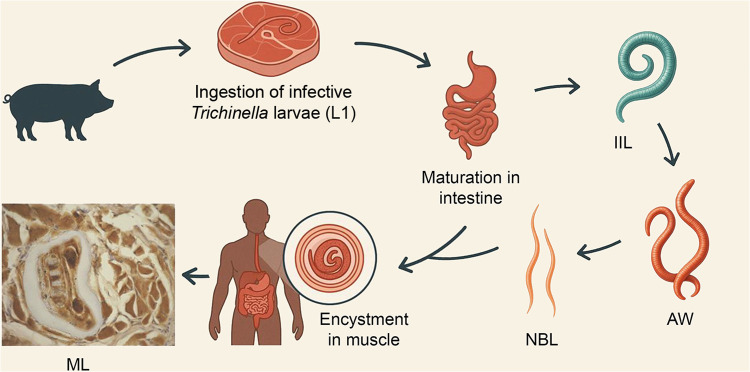
Life cycle of *Trichinella spiralis.* Abbreviations: IIL, Intestinal infective larvae; AW, Adult worms; NBL, Newborn larvae; ML, Muscle larvae; L1 (infective larvae) within the nurse cell. Immunohistochemical staining of ML was performed using *Trichinella*-positive human serum and peroxidase-labeled anti-human immunoglobulin G (HRP-IgG). The reaction was visualized with peroxidase chromogenic substrate 0.05% 3.3’-diaminobenzidine tetrahydrochloride (DAB) (magnification 40x)**.**

*Trichinella* spp. possess a unique characteristic among nematodes, which is a direct life cycle completed within a single host, involving intracellular stages in the intestinal epithelial cells (enterocytes) and skeletal muscle cells [6, Fig 1]. However, individual species differ in certain biological traits, such as encapsulation, host range, and environmental adaptations, which influence their epidemiology. Here we focus on the life cycle of *T. spiralis*, the species most relevant to human infection. Transmission typically occurs through the consumption of raw or undercooked meat from infected domestic or wild animals, with pork representing the primary source of *T. spiralis*-related trichinellosis worldwide. Ingested muscle larvae (ML) are liberated from the surrounding tissue under the action of gastric juices in the stomach, and through the influence of bile and pancreatic enzymes in the small intestine, develop into intestinal infective larvae (IIL). IIL invade the intestinal epithelium, i.e., rows of columnar epithelial cells, where they molt four times to grow into adult worms (AW), about 30 h post infection (p.i.) [7].

Sexual dimorphism is characteristic for nematodes of the genus *Trichinella*, with females (app. 3 mm in length) twice as large as males. Adults are covered with several cuticle layers, which protect inner organs during intestinal epithelium invasion. The male and female adult mate within gut mucosal epithelia and from day 4 p.i. females start to produce newborn larvae (NBL) [8]. A single adult female worm can produce as many as 500–1,500 NBLs (app. 0.08 mm in length), with the total number being influenced by the *Trichinella* species, host species, and the host’s immune status. NBLs are equipped with a sharp stylet placed in oral cavity, which serves to penetrate through membrane into the cells. NBL penetrate the submucosa and lamina propria, enter the lymphatic and circulatory systems, and are distributed throughout the body. Although they may be temporarily found in various tissues, only upon invading skeletal muscle cells they induce nurse cell formation (as a novel entity in the host body) and develop into infective ML, without molting. All species of the genus *Trichinella* nest in the nurse cell, but only those that encapsulate form collagen capsule surrounding the nurse cell [9]. Unlike most intracellular parasites, *Trichinella* does not kill the invaded muscle cell, and is therefore considered one of the most successful parasitic symbionts. A characteristic feature of the anatomy of *Trichinella* ML and AW is the exocrine organ known as stichosome, which consists of 45–55 large, stacked cells—stichocytes (ST). Stichocytes release excretory-secretory products (ES L1), crucial for establishing and maintaining the long-lasting host-parasite relationship [9].

## Epidemiology

Parasitic nematodes belonging to the genus *Trichinella* are globally distributed and infect a broad range of host species through both domestic and sylvatic transmission cycles. Spreading of *Trichinella* and epidemiology depend on the feeding behavior of host species and human influence [1,4,10]. The genus *Trichinella* includes two major clades distinguished by the presence or absence of a collagen capsule surrounding the larvae in muscle tissue. The encapsulated clade, which infects only mammals, includes seven species: *T. spiralis*, *T. nativa*, *T. britovi*, *T. murrelli*, *T. nelsoni*, *T. patagoniensis*, and *T. chanchalensis,* as well as three genotypes, *Trichinella* T6, T8, and T9, whose taxonomic status remains unresolved [11,12]. The non-encapsulated clade comprises *T. pseudospiralis,* which infects both mammals and avian hosts, and *T. papuae* and *T. zimbabwensis,* which infect mammals and reptiles*. T. spiralis* and *T. pseudospiralis* have a cosmopolitan distribution for two different reasons: *T. spiralis* has spread through human activities, whereas *T. pseudospiralis* disperses via migratory birds. The remaining taxa exhibit more restricted geographical ranges: *T. nativa* and *Trichinella* T6 occur in arctic and sub-arctic regions of North America, Greenland, and Russia; *T. britovi* in Europe, Western Asia, North, and West Africa; *T. murrelli* in United States of America, Southern Canada, and Northern Mexico; *T. nelsoni* in Eastern and Southern Africa; *T. patagoniensis* in South America; *Trichinella* T8 in Southwest Africa; and *Trichinella* T9 in Japan [13,14]. Unlike most *Trichinella* spp. that have been identified in registered outbreaks, genotype T8 and the species *T. zimbabwensis, T. patagoniensis*, and *T. chanchalensis* have not been associated with human infection. With the exception of *T. spiralis*, other *Trichinella* species primarily parasitize wild animals. The domestic and sylvatic cycles may function independently or interact, mainly as a consequence of human intervention. Improper management of domestic and wild animal populations can facilitate the transmission of certain *Trichinella* species (e.g., *T. britovi, T. pseudospiralis*) from the sylvatic to the domestic cycle, sometimes via synanthropic animals, such as rodents [8]. Domestic cycles occur on small farms with high-risk practices and uncontrolled housing conditions. On the other hand, industrial pork from big farms marketed internationally has never been found to be infected with *Trichinella*, and as such is safe for human consumption. Illegal hunting and import of *Trichinella*-infected pork, pigs, horses, and wild game, or their meat products, are usually the cause of family outbreaks [1,10,15].

A systematic review of the global epidemiology and clinical impact of human trichinellosis between 1986 and 2009 reported a total of 65,818 cases and 42 associated deaths worldwide, with the European Region accounting for 86% of all cases [16]. Low number of cases were recorded in the Americas, except in Argentina. In Africa, trichinellosis was documented only in Ethiopia, primary among Christian population, as well among Europeans in Algeria and Senegal. Sporadic cases also occurred in the Christian population of Lebanon and in Iran, largely linked to consumption of wild boar meat. Asian countries reported few outbreaks during this period, and several reports from China were excluded due to insufficient diagnostic details.

Most of the world’s trichinellosis outbreaks do not depend on the mere presence of *Trichinella* spp. among wild and domestic animals, but on cultural factors, i.e., eating habits (traditional consumption of raw or undercooked meat) that cause the transmission to humans [1]. Human eating habits, as well as practices of free-range animal husbandry, can increase exposure to *Trichinella*, especially in the regions where veterinary inspection does not exist or is not mandatory [17], or where it is avoided by farmers or hunters mostly for financial reasons [10]. In those parts of the world where prescribed control measures regarding farming and meat inspection are strictly enforced, trichinellosis becomes a rare disease, with the number of cases constantly declining. However, it still remains a risk because of the presence of *Trichinella* spp. in wildlife and potential spillover into domestic animals [1].

According to European Food Safety Authority and European Centre for Disease Prevention and Control (ECDC) reports for this century, the highest numbers of trichinellosis cases in Europe were associated with Bosnia-Herzegovina, Bulgaria, Lithuania, Poland, Romania, Russia, Serbia, and Spain. The latest ECDC annual epidemiological report for trichinellosis, which refers to year 2022, stated that 39 cases were recorded across 28 countries and an overall notification rate of 0.01 per 100,000 inhabitants, which represents a 49% decrease compared to the number of cases recorded in 2021. Latvia and Bulgaria had the highest notification rates in 2022, at 0.16 and 0.13 cases per 100,000 inhabitants, respectively. Notably, in 2023, Bulgaria, previously among the countries with the highest notification rates, reported no outbreaks of trichinellosis for the first time [18]. In contrast, ten years ago, in 2015, 29 European Union (EU)/European Economic Area countries reported 156 cases, which indicates that trichinellosis incidence in Europe shows a stable to decreasing trend with low notification rates, which is the result of compliance with all prescribed measures for controlled pig farming and inspecting meat intended for human consumption.

Yera and colleagues [17] presented the situation in Southeast Asia, where between 2001 and 2021, 1,604 cases of trichinellosis were reported in Cambodia, Laos, Malaysia, Thailand, and Vietnam. Most cases were concentrated in northern Laos (672 cases, *T. spiralis*) and northern Thailand (773 cases, *T. papuae*). No reported cases were noted in Myanmar and the Philippines during this period. China is one of a few of countries with the highest number of cases of trichinellosis in the world [19]. The high prevalence of trichinellosis in China is related to pig breeding and eating habits that include consumption of raw or under-cooked meat from wild animals that have not been inspected, since the testing of meat for *Trichinella* larvae is not mandatory.

In North America, trichinellosis is rare, while occasional outbreaks, such as one in North Carolina in 2023 [20], are associated with consumption of wild game meat. In South America, trichinellosis cases are associated mostly with Argentina and Chile [1,21]. In Africa, human trichinellosis has been reported only sporadically, with three confirmed outbreaks or case clusters documented in the 21st century, as summarized by Mukaratirwa and colleagues [22]. However, additional cases have been described in countries such as Algeria and Senegal, and the actual number of infections is likely to be underestimated, as the clinical picture can be misattributed to other parasitic diseases, including invasive schistosomiasis.

## Pathophysiology

Trichinellosis as clinical disease unfolds in biphasic pattern: intestinal (enteral) phase (time period between the larvae release from the cyst until the production of NBL) and systemic (parenteral or muscular) phase (associated with inflammatory and allergic responses caused by invasion of the skeletal muscle cells by the migrating NBL) [4]. In the intestinal phase, digesting meat with tissue cysts releases infective larvae (L1), which penetrate the mucosa of small intestine, occupying several columnar epithelium cells at the time, mature into adults, and start mating. By day 4 p.i., adult females begin to release a large number of NBLs. They cause transient inflammation by passing through organs like heart and brain before encysting in well-vascularized skeletal muscles such as the tongue, diaphragm, psoas, pectoralis major, and gluteus maximus [4]. The process of NBL release lasts for 2–3 weeks in humans, which depends on the intensity of the immune response initiated at the intestinal level that results in the expulsion of AW [8,23].

After entering muscle cells, NLB develop into L1, starting the muscle phase of the infection [8,24]. NBLs induce the transformation of muscle cells into nurse cells, degrading muscle proteins and causing structural changes. By day 8 p.i., contractile proteins and structural elements like myofibrils, Z-lines, I-bands, and A-bands are lost [25]. By day 12 p.i., nurse cell development is nearly complete, with the complete maturing by day 20 p.i. [26].

The ability of *Trichinella* to reside within host muscle cells without destroying them is crucial to its parasitic success. Its localization in skeletal muscle cells triggers morphological and biochemical changes, converting the infected cell into a nurse cell, a unique new type of host cells. Nurse cell formation results from a balance of degenerative and regenerative changes, showcasing the host-parasite interaction. The morphogenesis of the nurse cell is orchestrated through a two‑way host response: the infected myocyte de-differentiates, reenters the cell cycle, and then arrests at G2/M phase, while satellite cells activate, proliferate, and fuse, contributing to the eosinophilic cytoplasm that shapes the structure [24,25,27,28]. Infected muscle cells show upregulation of 184 genes related to differentiation, proliferation, and apoptosis [25]. Key myogenic transcription factors (MyoD, myogenin, Pax7, desmin, M-cadherin, Numb, MEF2, Pbx1, and NFAT) are significantly upregulated, coordinating transformation and balancing pro- and anti-apoptotic events in muscle cells [24,25]. As the infected myocyte reenters the cell cycle and arrests at G2/M, it develops up to ~100 markedly enlarged, hypertrophic nuclei with prominent nucleoli that cluster within the basophilic cytoplasm and give the nurse cell its characteristic appearance [24]. *Trichinella* ML may directly influence host gene expression by secreting factors, including small RNAs like microRNAs (miRNAs), which regulate gene activity [29]. Distinct stage-specific miRNA profiles across *T. spiralis* developmental stages highlight their role in the parasite growth and metabolism [30]. These miRNAs could be released from AW, ML, and NBL as a cargo of extracellular vesicles or in an extravesicular form in case of ML [29,31,32]. Moreover, miRNAs released by NBL, such as let‑7‑5p, modulate host immune cells by promoting the regulatory M2 macrophage phenotype and dampening inflammation, thereby facilitating immune evasion and enhancing larval survival [32].

**Fig 2:**
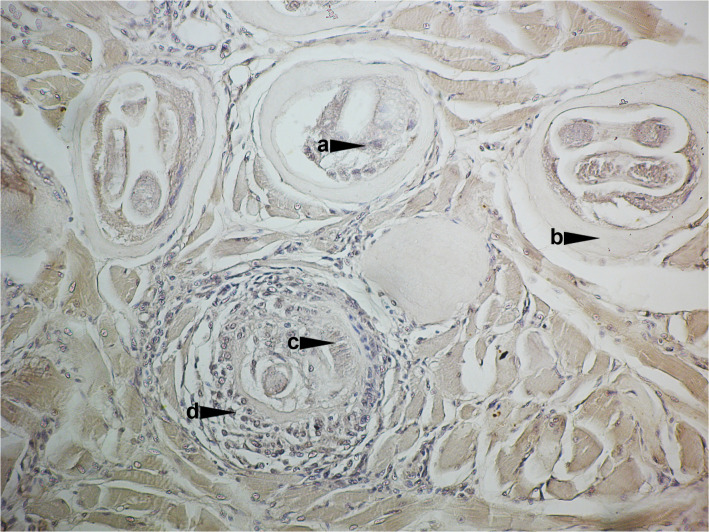
*Trichinella spiralis* larva encapsulated within a nurse cell in muscle tissue. Immunohistochemical staining was performed using *Trichinella*-positive human serum and anti-human HRP-IgG. The reaction was visualized with the peroxidase chromogenic substrate (DAB) (magnification 20×). Labels: **(a)** Nurse cell nuclei; **(b)** Collagen capsule; **(c)** Stichosome; **(d)** Inflammatory cells.

In encapsulated *Trichinella* species, infective muscle-stage larvae persist within a nurse cell enclosed by a collagen capsule mainly composed of collagen types IV and VI (Fig 2). Type IV, produced by the nurse cell, forms the inner layer and serves as a structural scaffold, while type VI, from surrounding fibroblasts, makes up the outer layer and supports cross-linking [9]. In non-encapsulated species, nurse cell is surrounded with a capsule which is incompletely formed due to limited satellite cell fusion [33]. To sustain themselves, larvae induce angiogenesis via cytokine-driven vascular endothelial growth factor release, forming a capillary network around the infected cell that ensures the delivery of nutrients and other small molecules and the release of larval products [25]. Glycogen accumulation aids survival, and the semi-permeable capsule blocks the entry of immune cells and immunoglobulins, creating an immune-privileged niche [34], where larvae can survive for years [1]. Calcification of encapsulated ML may begin after 6 months; however, it does not occur in all parasites at the same time. It is assumed that the timing depends on the location of the parasite within the host, as well as on the species of *Trichinella*, the host species, and the host’s immune response. The larvae enter a hypobiotic state in which they remain until their death or until ingested by another host [4]. Another adaptation that ensures persistence of larval viability is anaerobic metabolism of the larvae [9], which favors its survival in decaying carcasses.

**Fig 3:**
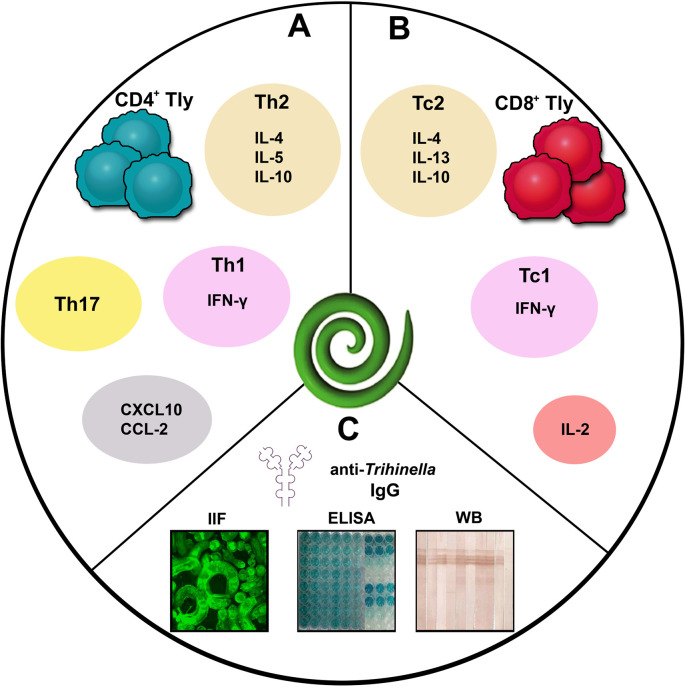
Immune responses triggered during the muscular phase of trichinellosis. **(A)** Cytokine and chemokine production by *Trichinella*-specific CD4^+^ T cells; **(B)** Cytokine production by *Trichinella*-specific CD8^+^ T cells; **(C)** Serological tests for the diagnosis of trichinellosis—detection of anti-*Trichinella* IgG antibodies. Abbreviations: Tly, T lymphocyte; Th1, T helper type 1 cell; Th2, T helper type 2 cell; Tc1, Type 1 cytotoxic T lymphocyte; Tc2, Type 2 cytotoxic T cells; IFN-γ, Interferon gamma; IL-4, Interleukin-4; IL-5, Interleukin-5; IL-10, Interleukin-10; IL-13, Interleukin-13; IFN-γ, Interferon gamma; CXCL10, Chemokine (C-X-C motif) Ligand 10; CCL-2, C-C motif ligand 2; IgG, Immunoglobulin G; IFA, Indirect Immunofluorescence Assay; ELISA, Enzyme-Linked Immunosorbent Assay; WB, Western Blot.

ML of the genus *Trichinella* maintains interaction with the host via its ES L1 products, a mixture of diverse proteins, glycans, lipids, nucleic acids, and extracellular vesicles [6,35–37]. Most of the proteins are glycosylated, bearing multiantennary N-glycans capped with tyvelose (3,6-dideoxy-D-arabinohexose) [9]. Tyvelose is an immunodominant carbohydrate epitope, which triggers strong antibody responses that prevent reinfection. Anti-tyvelose antibodies aid in parasite expulsion by blocking attachment to enterocytes and hindering intestinal niche formation [38]. ES L1 products comprise a diverse array of functional proteins, including heat shock proteins, endonucleases, proteinases, protein kinases, proteinase inhibitors, superoxide dismutase, glycosidases, as well as extracellular vesicles, which participate in the induction of the immune response [6,35–37]. The knowledge regarding immune mechanisms triggered during different stages of *Trichinella* infection has been mostly gained from experimental mice and rat models, whereas data for human infections primarily concern antibody responses. Characterization of cell-mediated immunity in trichinellosis originates from a limited number of studies based on specific stimulation of peripheral blood mononuclear cells with *Trichinella* ML antigens and subsequent analysis of T-cell subsets (Fig 3). These findings indicate that the muscle phase of infection in humans elicits a mixed Th1/Th2 response, with a predomination Th2 profile, and the presence of Th17 cells [39–42]. Moreover, ES L1 products also participate in orchestrating complex immunomodulation, including suppression of inflammation, complement evasion, modulation of dendritic cell activity [6,43–46]. Insights from *in vitro* and *in vivo* studies on the immunomodulatory roles of *Trichinella* ES L1 products may contribute to the development of novel therapeutic approaches for allergies and autoimmune diseases [6,47,48].

## Clinical manifestations

Symptoms of trichinellosis typically appear 1 to 4 weeks following the consumption of infected meat, with an incubation period of 7–30 days, shorter in more severe infections due to higher larval burden [25]. Disease severity largely depends on the inoculum size, which is difficult to quantify, as well as on the *Trichinella* species and host factors such as age, gender, ethnicity, and immune status [23]. The classic triad of signs and symptoms, fever, periorbital edema, and muscle pain, is frequently observed and should be carefully assessed by medical practitioners when trichinellosis is suspected.

Early symptoms are nonspecific: malaise, headache, diarrhea, and gastrointestinal discomfort, often accompanied by high fever (39–40 °C) lasting 8–10 days [49]. The intestinal phase begins around 2 days p.i. with gastroenteritis caused by IIL invading the intestinal mucosa. Additional symptoms such as anorexia, nausea, vomiting, abdominal pain, and constipation may also occur, with diarrhea generally being more prolonged than vomiting [23]. The subsequent parenteral phase occurs as released NBLs migrate to skeletal muscles, provoking systemic symptoms. During trichinellosis, *T. spiralis* releases stage-specific antigens that activate dendritic cells and initially elicit a Th1 response characterized by IL-12, IFN-γ, TNF-α, IL-1β, and nitric oxide (NO), contributing to intestinal inflammation. As larvae disseminate, the response shifts toward a predominant Th2 profile, marked by increased IL-4, IL-5, IL-10, and IL-13, which promote eosinophil and mast cell proliferation, IgE production, and facilitate parasite expulsion [6]. This Th2 polarization explains the marked infiltration of eosinophils, mast cells, monocytes, and lymphocytes into affected tissue. IL-5 in particular drives eosinophil maturation and recruitment, making eosinophilia a characteristic finding in trichinellosis [23]. Mast cell degranulation, and the release of mediators such as histamine, prostaglandins, and bradykinin contribute to capillary leakage and the development of characteristic facial or periorbital edema [4]. Because *T. spiralis* matures and reproduces rapidly, both Th1 and Th2 responses overlap, with Th2 dominance emerging during NBL dissemination. In the muscle phase, regulatory T cells accumulate near infected tissue, likely reflecting activation of regulatory pathways, and inflammation gradually subsides and may resolve completely. Throughout their life, *Trichinella* ML, through their ES L1 products, modulate host immune responses triggered by additional host or environmental factors, thus contributing to the restoration of homeostasis [6]. Clinical manifestations at parenteral stage include fatigue, myalgia, and conjunctivitis. Periorbital and facial edema, as well as cutaneous rash, are considered IgE-mediated allergic manifestations associated with trichinellosis [50]. Additional signs may include rash, petechiae, subungual hemorrhages, and ocular muscle pain [4]. Myalgia varies in intensity based on disease severity and typically involves the trunk and limbs. In severe cases, patients may experience significant muscle weakness or disability due to lesions like angiomyositis and neuromuscular complications [49].

The clinical course of trichinellosis varies widely; it may be asymptomatic, abortive, mild, or moderate to severe, sometimes with complications [4, Table 1]. Most cases resolve within a few months, especially with prompt treatment, but complications can occur, occasionally in severe or untreated infections, and in the older adults. Ocular muscle invasion may cause eye pain, diplopia, paralysis, or accommodation issues [49]. Serious complications involve the cardiovascular system (e.g., myocarditis, pericardial pain, electrocardiogram changes) and respiratory system (e.g., dyspnea, pneumonia, bronchitis) [51,52]. Pulmonary manifestations may include cough, shortness of breath, and patchy infiltrates visible on the chest X-rays [53]. Dyspnea is relatively frequent respiratory symptom, typically resulting from parasitic invasion of the diaphragm and inflammation of the respiratory muscles [49,54]. Late-stage pneumonia and pleuritis may occur due to bacterial infection [4]. Neurological complications, though rare, include meningitis, encephalopathy, and cortical infarcts [49]. In some cases, focal brain lesions can occur, most commonly presenting as motor deficits, particularly hemiparesis, while involvement of cerebellum and cranial nerves is less frequently observed [55].

**Table 1:** Clinical spectrum of trichinellosis.

Trichinellosis phase	Clinical features
Intestinal phase (1–2 days postinfection)	Abdominal pain, diarrhea, nausea, vomiting
Systemic phase (1–2 weeks postinfection)	High fever, periorbital/facial edema, myalgia, eosinophilia
Severe complications	Myocarditis, pneumonia, encephalitis (rare, life-threatening)
Chronic manifestations	Persistent fatigue, reduced muscle strength, long-term myalgia

In some individuals, trichinellosis may progress to a chronic form characterized by persistent fatigue, myalgia, reduced muscle strength, or ocular disturbances that can persist for years or even decades [56,57]. This condition is associated with the continued presence of IgG antibodies in the serum, electromyographic abnormalities, and infiltration of inflammatory cells in muscle tissue [58,59]. It is referred to as either chronic trichinellosis or as a sequelae of the acute phase [49].

## Diagnosis

Diagnosis of trichinellosis can be particularly challenging in cases with atypical clinical presentations or in sporadic cases that present with nonspecific symptoms such as leukocytosis with eosinophilia, fever, fatigue, and myalgia, which may indicate diseases other than trichinellosis [4,60]. Therefore, diagnosis is based on three main criteria issued by the European Center for Disease Control: a patient’s history and relevant epidemiological data, clinical examination, and laboratory findings, which include serological testing for the presence of anti-*Trichinella* antibodies. Definitive diagnosis could be made by muscle biopsy [49]. However, muscle biopsy is both invasive and painful, and therefore often avoided, especially since it may not always yield definitive results, even when clinical suspicion of trichinellosis is well-founded, as it often gives negative results during the early phase of the disease. Diagnostic sensitivity of muscle biopsy increases several weeks after infection [23]. It is only used in severe cases when it is impossible to reach a conclusion based on serological findings and differential diagnosis. It should be pointed out that muscle biopsy enables species identification, using methods based on polymerase chain reaction, which is important in cases when the source of the infection could not be traced back. In addition, in situations where the etiological agent remains unclear, western blot analysis using *T. spiralis* crude worm extract can assist in distinguishing infections caused by encapsulated versus non-encapsulated *Trichinella* species [61], while new approaches based on cellular immune-response profiling may further support species differentiation [41].

For an accurate diagnosis, it is necessary to collect detailed medical history of the patient and determine the behavior (especially eating habits) of the patient in the period coinciding with a possible *Trichinella* infection. Identifying an epidemiological link, such as exposure to a common infection source or consumption of contaminated meat, is a key factor in considering trichinellosis outbreak as part of the differential diagnosis [52]. Laboratory findings such as leukocytosis, eosinophilia, elevated levels of muscle enzymes (e.g., creatine phosphokinase, lactate dehydrogenase, transaminases, and aldolase), and increased total IgE are considered nonspecific for trichinellosis, but still may help in determining the diagnosis [4,23,49,52, Table 2]. Serological testing, typically involving the detection of specific immunoglobulins in the serum, on the other hand, provides valuable diagnostic support. The International Commission on Trichinellosis recommends the use of the ELISA test with ES L1 antigens for the serological diagnosis of *Trichinella* infection in humans [61,62]. Compared to crude somatic ML extract, ES L1 antigens show limited cross-reactivity with antibodies produced against other parasitic infections. Additional serological methods such as indirect immunofluorescence assay (IFA), indirect hemagglutination, and western blot are also utilized (Fig 3C). Serological tests are often negative in the early stages of infection, therefore retesting after the initial sample is recommended, and evidence of seroconversion provides strong diagnostic value. To achieve reliable diagnosis, it is advised that at least two different tests using two different antigens (i.e., ES L1 in ELISA and whole ML in IFA) should be performed in diagnostic laboratory [23]. IFA using whole larvae as an antigen can detect specific immunoglobulins earlier than ELISA, a week after appearance of trichinellosis symptoms [54,60]. However, the disadvantage of this method is its potential cross-reactivity with antibodies from other parasitic infections or autoimmune diseases, which may lead to false-positive results. Therefore, western blot should be used as a confirmatory test for ELISA findings, especially in cases where the results are inconclusive, to avoid both false-negative and false-positive outcomes.

**Table 2:** Diagnostic scheme for estimating the probability of trichinellosis (created according to data interpretation from Refs. [49] and [52]).

Group of clinical and laboratory criteria	
a. Major clinical signs	• Fever • Eyelid and/or facial edema • Myalgia
b. Additional clinical signs	• Diarrhea • Neurological signs • Cardiological signs • Conjunctivitis • Subungual hemorrhages • Cutaneous rash
c. Laboratory findings	• Eosinophilia (>1,000 cells/µl) and/or increased total IgE • Elevated muscle enzymes
d. Parasitological/serological findings	• Positive serology (highly specific test) • Seroconversion • Positive muscle biopsy
Diagnostic interpretation of trichinellosis: Very unlikely: 1a/1b/1c Suspected: 1a/2b + 1c Probable: 3a + 1c Highly probable: 3a + 2c Confirmed: 3a + 2c + 1d or any of a/b + 1c + 1d	

Early and accurate diagnosis is a prerequisite for timely and effective treatment. Seroconversion happens between 2nd and 3rd week p.i. [63]. During the early phase of infection, anti-*Trichinella* antibodies are primarily directed against antigens of IIL and AW, which may include stage-specific epitopes absent from the ES L1 products commonly used in ELISA assays. Consequently, serum samples collected during the window period between *Trichinella* infection and detectable antibody levels may yield false-negative results. Since ES L1-based ELISA lacks sufficient sensitivity for detecting antibodies in the early stages of infection, attempts have been made to construct a test using parasite components from IIL and AW. Antigens from IIL, which are the first to interact with host immune cells, may serve as important early markers of trichinellosis. Sun and coworkers [64] demonstrated that ELISA based on IIL excretory-secretory products could detect anti-*Trichinella* antibodies in patients as early as 19 days p.i. Similar results were obtained when sera of patients with trichinellosis at 19 days p.i. were tested using ELISA with AW ES [65]. Although the sensitivity and specificity of tests with IIL and AW ES antigens exceeded ELISA with ES L1, the process of isolating IIL and adults from the intestine for antigen preparation is much more demanding than obtaining ML.

## Treatment

Treatment of trichinellosis is based on patients’ symptoms, anamnestic data (history of consuming raw or undercooked meat that has not been tested for *Trichinella*), and laboratory findings.

Initial symptoms of clinically manifested infection, such as headache and fever, are usually treated symptomatically with antipyretics and anti-inflammatory drugs. *Trichinella* infection with systemic complications is treated with antiparasitic drugs (albendazole 400 mg twice a day per orally for 8–14 days, mebendazole 200–400 mg three times a day for 3 days, then 400–500 mg three times a day for 10 days) and corticosteroids (prednisone 30–60 mg daily for 10–15 days). Albendazole and mebendazole are not considered as safe for use in pregnant women and children under 2 years of age [49]. For children older than 2 years, albendazole is administered at a dose of 5 mg/kg body weight twice daily for 10–15 days*.*

Confirming the diagnosis enables the early initiation of antiparasitic therapy, which is essential for preventing or alleviating trichinellosis symptoms. The major limitation of delayed treatment lies in the low susceptibility of migrating and encapsulated ML to anthelmintic agents [4]. According to Faber and colleagues [66], antiparasitic drugs are effective when administered within the first 6 days post-exposure, during the phase of intestinal invasion by IIL, when anthelmintics primarily act by expelling worms from the intestine. Three comparative studies assessing treatment efficacy have shown that mebendazole and albendazole should be considered as the first-line therapies for the acute phase of trichinellosis [52]. However, prospective, randomized studies are still urgently needed to establish optimal treatment regimens for systemic trichinellosis [67]. Moreover, early diagnosis of trichinellosis is uncommon, and most patients are identified several weeks postinfection, by which time larvae have already established within muscle cells.

## Prevention

Trichinellosis remains endemic in parts of World [4], largely due to insufficient awareness among small-scale and backyard pig farmers, who often neglect hygiene standards and fail to submit meat for veterinary inspection. Uninspected pork is sometimes shared informally or sold on local markets, facilitating further spread of the infection. Exported cases have been documented, such as simultaneous outbreaks in Serbia and France [68]. Emerging risks include infections acquired during wildlife hunting tourism in North America or through consumption of exotic meats, such as polar bear in Greenland [69,70]. Traditional food practices like consumption of raw or undercooked meat, homemade sausages, or products prepared by smoking, fermenting, or drying of uninspected meat, also contribute to transmission, as these methods may not reliably inactivate *Trichinella* larvae [54,60,71]. Wild boar meat infected with *T. britovi* is another significant source of infection when not properly tested before consumption [72–74]. Conversely, countries with advanced veterinary infrastructure have drastically reduced pork-related trichinellosis through high-biosecurity farming and routine meat inspections [3,75]. EU Regulation No. 2015/1375 mandates *Trichinella* testing for slaughtered pigs, wild boars, horses, and other susceptible animals, excluding pigs slaughtered for private use. With pork-related cases declining, infections from wild game meat are now of growing concern [2].

Eating untested raw and undercooked meat should be avoided. The most important prevention measure is to make sure that pork and pork products and meat from wild animals are properly cooked to safe temperatures (71 °C in the middle of small pieces of meat) before consumption. Salting, drying, and smoking pork meat does not consistently kill infective *Trichinella* larvae, so homemade sausages made from infected meat were the cause of cases of trichinellosis reported worldwide in recent years (FAO, 2014; https://www.fao.org/family-farming/detail/en/c/273649/). The United States Department of Agriculture recommends freezing meat less than 15 cm thick for 20 days at ‒15 °C to kill *Trichinella* larvae. However, freezing game meat is not safe enough because some *Trichinella* species that infect wild animals are freeze-resistant, and freezing will not effectively kill all the larvae. Although microwave cooking is widely used for its convenience, its safety in inactivating *Trichinella* larvae remains uncertain, as uneven heat distribution may leave portions of meat undercooked; therefore, additional research is needed before microwave cooking can be considered a reliable method for preparing potentially infected pork [76].

*Trichinella* control measures include pig identification, mandatory testing of all pigs slaughtered for human consumption, surveillance of wild boar populations, rodent control on pig farms, and monitoring of these preventive actions. Collectively, these measures have a One Health impact, reducing the prevalence of infection in animals and, consequently, the number of human trichinellosis cases. Public awareness campaigns through the media are necessary to inform consumers about the risk of trichinellosis. Although implementation of veterinary measures has resulted in a reduction of *Trichinella* infections in pigs, the existence of outbreaks worldwide indicates insufficient public awareness and suggests that further efforts should be made to continue education and strengthening of preventive measures [10].

EU regulations require that veterinarians and laboratory staff that inspect meat for the presence of *Trichinella* spp. larvae must be properly trained and use officially recommended methods for detection (ISO/IEC, 2015, and European Union, 2015). Further, EU laboratories in charge of official controls must regularly participate in Proficiency Testing (PT) schemes for the detection of *Trichinella* larvae in meat by artificial digestion [2,77], and some non-EU countries (like Serbia) participate also [78–80]. Continuous participation in PT schemes increase performance of participating laboratories by positively affecting the staff’s accuracy in sample testing by artificial digestion [78,80].

For better prevention of trichinellosis, we need: (a) Education of consumers of backyard pigs and game meat about the risk, as well as producers and hunters; (b) A functional quality assurance system in laboratories that perform official *Trichinella* testing and regular participation in PT. Cooperation at the global level, like the European network of parasitological laboratories (National Reference Laboratories) organized by the Istituto Superiore di Sanità (European Reference Laboratory for Parasites, ISS, Rome, Italy), proved to be very useful.

Although advances in the identification and characterization of stage-specific and immunomodulatory *Trichinella* proteins have generated interest in vaccine development [81,82], the practical applicability of vaccination in domestic pigs or humans remains limited. Under modern, well-managed husbandry conditions, the risk of *T. spiralis* infection in livestock is generally low, reducing the justification for routine vaccination of pigs. In humans, trichinellosis could be associated with consumption of insufficiently cooked meat from wild game; moreover, infections may involve not only *T. spiralis,* the most common etiological agent, but also *T. britovi* and other *Trichinella* species, making the development of a broadly effective human vaccine challenging. Nevertheless, the ongoing identification and characterization of stage-specific and immunomodulatory *Trichinella* proteins continue to provide promising starting points for vaccine research, leaving open the possibility of creating effective next-generation vaccines based on recombinant proteins, DNA, or viral vectors as part of preventive strategies [81].

## Future prospects

The future of trichinellosis research will, on one hand, continue to focus on reducing global incidence through improved hygiene, better livestock management practices that minimize pigs’ exposure to infection, stricter inspection protocols, and public education on safe food preparation and meat inspection. On the other hand, emerging technologies will provide new insights into the parasite’s life cycle, deepen our understanding of the immune response, and shed light on host–pathogen interactions, knowledge that may ultimately contribute to the development of novel therapeutic approaches for chronic inflammatory diseases.

### Key learning points

**Table:** 

1	**Transmission and Life Cycle**—Trichinellosis is acquired by eating raw or undercooked meat (pigs, wild animals) containing infective larvae; the infection progresses from an intestinal phase to systemic larval migration into striated muscles resulting in reprogramming of myocytes into nurse cells, a unique and novel host cell type that supports larval survival, enabling long-time persistence.
2	**Clinical Manifestations**—Symptoms range from mild gastrointestinal issues to systemic illness (fever, periorbital edema, myalgia), with some cases developing chronic fatigue and muscle weakness.
3	**Immune Response**—The disease is associated with a Th2-dominated immune response and marked eosinophilia.
4	**Diagnosis and Prevention**—Diagnosis relies on clinical signs and symptoms, laboratory findings, and link with epidemiological data, prevention focuses on meat inspection, controlled farming, and public education.
5	**Public Health Relevance**—Despite progress in food safety and research, trichinellosis remains a public health concern in endemic regions, highlighting the need for better understanding of disease mechanisms and species-specific differences.

### Selected publications

**Table:** 

1	Wu Z, Nagano I, Takahashi Y. *Trichinella*: what is going on during nurse cell formation? *Vet Parasitol*. 2013; 194: 155–9. https://doi.org/10.1016/j.vetpar.2013.01.044 [26]
2	Bruschi F, editor. *Trichinella* and Trichinellosis. London, UK: Academic Press; 2021 https://doi.org/10.1016/C2019-0-02594-0 [1,12]
3	Bruschi F, Ashour DS, Othman AA. *Trichinella*-induced immunomodulation: Another tale of helminth success. *Food Waterborne Parasitol*. 2022; 27: e00164. https://doi.org/10.1016/j.fawpar.2022.e00164 [21]
4	Bruschi F, Gómez-Morales MA, Hill DE. International Commission on Trichinellosis: Recommendations on the use of serological tests for the detection of *Trichinella* infection in animals and humans. *Food Waterborne Parasitol*. 2019; 5(14):e00032. https://doi.org/10.1016/j.fawpar.2018.e00032 PMID: 32095603 [60]
5	Gamble HR. *Trichinella spp*. control in modern pork production systems. *Food Waterborne Parasitol*. 2022; 28:e00172. https://doi.org/10.1016/j.fawpar.2022.e00172 PMID: 35942058 [75]
